# Applying the photovoice method with adolescents in mining areas in rural Mozambique: critical reflections and lessons learned

**DOI:** 10.1080/16549716.2024.2305506

**Published:** 2024-02-07

**Authors:** Olga Cambaco, Hermínio Cossa, Andrea Farnham, Eusébio Macete, Mirko S. Winkler, Karin Gross, Khátia Munguambe

**Affiliations:** a Epidemiology and Public Health Department, Swiss Tropical and Public Health Institute (Swiss TPH), Allschwil, Switzerland; b University of Basel, Basel, Switzerland; c Population Studies Unit, Centro de Investigação em Saúde de Manhiça (CISM), Maputo, Mozambique; d Department of Public Health & Global Health, Division of Infectious Diseases, Epidemiology, Biostatistics and Prevention Institute, University of Zurich, Zurich, Switzerland; e Community Health Department, Faculty of Medicine, University Eduardo Mondlane (UEM), Maputo, Mozambique

**Keywords:** Adolescent, focus group discussion, health and well-being, mining areas, Mozambique, participatory research, photovoice

## Abstract

There is a recognised need for innovative methods to elicit the perspective of adolescents on public health issues, particularly when addressing sensitive topics such as the impact of mining projects on their health. Participatory approaches such as “photovoice” allow for deep engagement of vulnerable and marginalised populations, including adolescents. However, few existing studies have used the photovoice method to reflect on issues related to the environment and its impact on public health. To date, no studies have been found that have used photovoice to gain insight into adolescentsʼ perspectives in mining areas. In this paper, we discuss the application of the photovoice method to understand adolescents’ perceptions about the impact of mining on their health and well-being in rural areas in Mozambique. The study was conducted in northern and central Mozambique. Photovoice was successfully integrated into eight focus group discussions with adolescent girls and boys aged 15 to 17 years. Several lessons for guiding future research were learned. First, it provided an understanding of the perceived impacts of mining on their health and well-being. Second, photovoice promoted active engagement and interest in the study by the adolescents. Finally, compared to its ability to capture perceptions of physical and environmental aspects affecting adolescents’ well-being, the method was less straightforward in revealing their concerns regarding social, relational and community aspects that are less tangible. Programs can make use of photovoice to address health issues without setting adolescents’ views and priorities aside, allowing them to influence health decisions on issues that are meaningful to them. Future studies should explore strategies to minimise the role of the power dynamics that affect the engagement and contribution of adolescents in advocating for necessary and meaningful changes. Additionally, it is important to investigate how health programs and policies can help to reduce the impact of existing inequalities.

## Introduction

Photovoice is a method used to elicit visual representations of participantsʼ perspectives and everyday realities [1]. Wang and Burris, who developed this method in the 1990s, defined ‘photovoice’ as *‘a process by which people can identify, represent and enhance their community through a specific photographic technique’* [2]. They used the method to support rural Chinese farmers in documenting their daily health and working conditions and identifying desired public health improvements in the context of their everyday life [2,3]. The photovoice method has its roots in three relevant theoretical underpinnings: Freire’s education for critical consciousness theory, feminist theory, and a community-based participatory approach to documentary photography [2–4]. Today, photovoice is part of a large group of visual methodologies (e.g. photojournalism, documentary photography, participatory photography, photo-elicitation, digital storytelling and photo story) that use images as data [5–8].

Photovoice enables individuals and communities to identify, reflect on and represent their experiences and concerns, which can then be communicated to different stakeholders, including policymakers, in an easily accessible way [9]. In practice, it involves providing participants with cameras in order for them to take photos related to a specific topic, representing personal or community experience in that domain [10]. The photos are then used to promote a critical dialogue in which participants reflect on their experiences and take a critical stance on their photographs [11]. This reflection is guided by a method called SHOWeD, helping participants build captions for their photos based on the following five questions: (i) what do you SEE here? (ii) what is really HAPPENING here? (iii) how does this relate to OUR lives? (iv) WHY does this situation exist? and (v) what can we DO about it? [12].

Photovoice has gained a broad application across disciplines, including education, environmental health, public health, social science and sports. Its expansion across disciplines can be attributed to the flexibility, adaptability and participatory characteristics of this method, as described in previous studies [13,14]. Additionally, photovoice application has been determined by factors such as the project’s objectives, available resources, project duration and the influence on decision-making process [1,15]. However, its use in different settings and disciplines has raised concerns regarding the rigour of the method and its lack of standardisation, with scholars often following the photovoice steps arbitrarily [16,17].

Photovoice has also been applied to a variety of populations and age groups to capture individual and/or community experiences [16]. The method has particularly become a strong tool for empowering people who are frequently disregarded or undervalued [1,5,11,13,18]. The photovoice method is also employed for (i) communication, (ii) education, (iii) exploration, and (iv) awareness [1]. The photovoice has been successfully applied to vulnerable and marginalised populations to promote their health and well-being [1,19,20]. Qualitative health-related research and projects involving adolescents have applied photovoice in different settings on topics such as (i) substances and tobacco use, (ii) barriers to health care, (iii) racism and other forms of discrimination, (iv) obesity/body image, (v) violence, (vi) mental health, (vii) suicide, and (viii) food environment [1]. However, despite the usefulness of this method in addressing stratified community subgroups in remote settings, to date, no studies have been found that have used photovoice to gain insights from communities affected by mining projects, and particularly for adolescents.

While mining projects have been expanding in many African countries and might contribute to their economic growth, health-related studies addressing the influence of mining on the health of community subgroups, including adolescents in SSA countries remain scarce [21,22]. The existing literature provides evidence for their impact on local population health, migration patterns, and economic and social development [21,23]. Additionally, an increasing number of studies report adverse health impacts of mining projects on different population groups in mining areas in both, the environment and its social determinants [21,23–25]. These studies suggest that adolescents growing up in mining-affected communities are affected by a range of health and social impacts [21,26,27]. This also applies to adolescents being exposed to negative health impacts in several ways such as environmental health hazards, drop school out, high levels of in-migration, experience of sexual violence, substance abuse, sex work, and contract sexually transmitted infections (STIs), including HIV [28–31]. Particularly, adolescents that face unprecedented challenges, encompassing the complex burden of diseases of poverty and inequalities [27,32,33].

Against this background, novel research approaches are needed to gain a better understanding of adolescentsʼ experiences in mining project areas and the impact on their health from the perspective of adolescents themselves, as well as for identifying the most pressing health and well-being issues [21]. In this paper, we discuss the application of the photovoice method to understand adolescents’ perceptions about the impact of mining projects on their health and well-being in rural areas in Mozambique. Additionally, a reflection on lessons learned in applying the method is presented. Comprehensive findings generated by the full group discussions and the analysis of the photos generated by photovoice participants are discussed in detail in a separate publication.

## Methods

### Study design

This paper is based on a qualitative research study investigating the impact of mining activities on the health and well-being of adolescent girls and boys aged 13 to 17 years living in mining areas in Mozambique. The study employs a combination of qualitative data collection techniques, namely: (i) non-participant observations, (ii) photovoice integrated in focus group discussions (FGDs) with adolescents, (iii) standalone FGD with caregivers and key (iv) informant interviews with stakeholders working and/or currently involved and/or participated in adolescent health. The results and analysis presented in this paper are drawn from the photovoice method, which was incorporated into eight FGDs with adolescents aged 15 to 17 years.

### Study site

**Figure 1.**
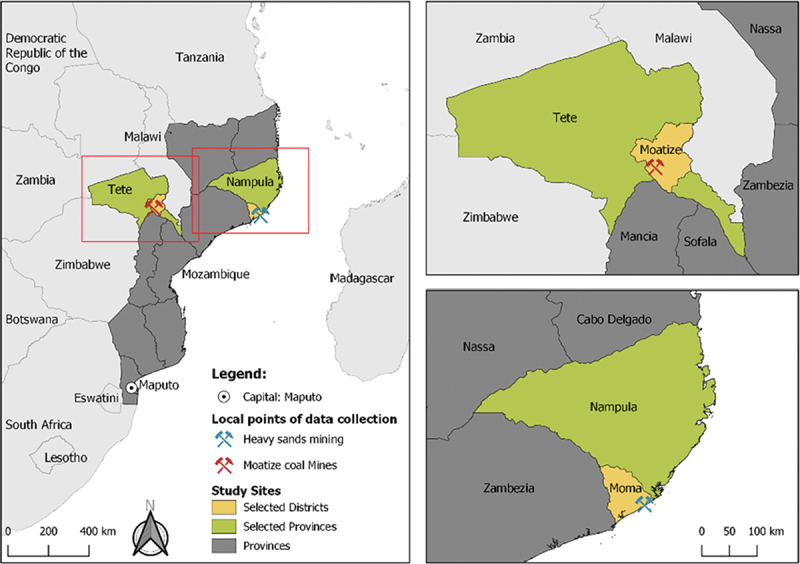
Study sites map and type of respective extracted commodities.

The present study took place from May to July 2022 in northern and central Mozambique in the districts of Moma and Moatize, located in the provinces of Nampula and Tete, respectively (Figure 1). The two sites were purposely selected based on the presence of mining projects in the communities. These rural districts have a low urbanisation rate, and the principal occupations of the community members are agriculture, forestry, fishery, livestock, trading, and handicrafts [34]. According to the National Institute of Statistics, the population in Moma was 349,864 inhabitants (49.4% female and 50.6/male) and in Moatize 292,341 (51.3% female and 48.7% male). While Islam is predominant in Moma district, Catholicism is the dominant religion in Moatize. In both districts, the leading causes of morbidity and mortality are malaria, HIV/AIDS, sexually transmitted diseases (STDs), tuberculosis, and diarrhoeal diseases [35,36]. The study was conducted in four neighbourhoods in Moma (Pillivili A, Pilivili C, Hori, and Namalico) and three neighbourhoods in Moatize (1° de Maio, Nhanchere and 25 de Setembro) (Table 1).

**Table 1.** Target population - focus group discussions with adolescents aged 15–17 years old.

District	Target population	Neighbourhood	Number of participants	Number of photographers
Moma district	Adolescent resident girls without children/not pregnant	Pilivile Sede A	6	3
	Adolescent resident boys*	Pilivili C	12	3
	Adolescent migrant boys	Hori	10	3
	Adolescent resident girls with children/pregnant	Namalico	9	3
Moatize district	Adolescent resident girls without children/not pregnant	1° de Maio	9	3
	Adolescent resident boys	Nhanchere	9	3
	Adolescent migrant boys	25 de Setembro	7	3
	Adolescent resident girls with children/pregnant	Liberdade	8	3

*It is more common to find adolescent migrant boys in mining areas due to the nature of the jobs and activities they seek as migrants in such settings [37–40].

### Study population

In each of the districts, the study population comprised of adolescent girls and boys aged 13 to 17 years old living in close proximity to mining sites. In order to minimise the burden on very young adolescents (13 and 14 years old), the photovoice technique integrated into the FGDs was conducted with 15- to 17-year-old adolescents only, taking into account that this group is more likely to handle the camera responsibly and consciously regarding sensitive and ethical aspects.

### Field work set-up

Field activities were led and co-ordinated by the Principal Investigator (PI) trained in social sciences (OC), supported by an environmental epidemiologist (HC) and a senior socio-behavioural health researcher (KM). At the local level, the PI coordinated the activities of the gender-balanced team, consisting of two community guides, four interviewers, and four transcribers per district, all with prior experience in working with community members, particularly adolescents. The main facilitators for the FGDs were young people who were fluent in the local languages. Prior to data collection, team members were trained for 1 week at each site in good clinical practices, study protocol, study-specific procedures for conducting FGDs, instruction in the photovoice technique and ethical considerations. Procedures were piloted before study implementation in order to test the data collection guides to conduct photovoice integrated into FGDs.

### Community entry

Authorisation for conducting the study was obtained from District Health Services, Municipal Councils, and local community leaders in both districts. Moreover, introductory meetings were organised with local government representatives at the district level to explain the objective of the study and to share the fieldwork activity plan and receive specific recommendations for adapting the study procedures to the local context, where appropriate. Prior to the data collection, transect walks were conducted with the assistance of the local community guides. This allowed the researchers to obtain an overview of the study site and to develop a map of the communities’ neighbourhoods, mining company set-up, proximity of houses to mines and typical adolescents’ hanging out places (e.g. place of work, markets, health facilities, mines, football fields, etc.). This approach helped to understand the context in which adolescents, their families and communities live, to ensure the most appropriate recruitment strategy and data collection approach.

### Recruitment of study participants

Participants were purposively sampled based on specific criteria related to the study objectives [41]. With the help of the community leaders, between six and ten eligible adolescents aged 15 to 17 years were identified in each neighbourhood and invited to participate in the FGDs. In order to avoid power imbalances while assuring the diversity of participant views in each site, FGDs were stratified by sex (male/female), maternity status (pregnant/mother of children/no children), and place of origin (resident/immigrant) (Table 2). To ensure an inclusive selection process, community leaders were instructed to also consider the most vulnerable and/or marginalised adolescents, namely married, disabled, or orphaned, and not to suggest the selection of their own close relatives. In addition, participants were selected from different households to minimise clustering of ideas and opinions. For each FGD, the research assistants, together with the community leaders, selected three adolescents to act as photographers based on their technical affinity, enthusiasm, and proactivity. None of the photographers had previously owned or used a digital camera before or participated in a previous study.

**Table 2.** Participants’ sociodemographic profile.

Characteristics of participants	Moatize (*N* = 33)		Moma (*N* = 47)		Total (*N* = 80)	
	n	%	n	%	n	%
**Age**						
15 years	6	18.2	12	25.5	18	22.5
16 years	11	33.3	11	23.4	22	27.5
17 years	16	48.5	24	51.1	40	50
**Sex**						
Male	17	51.5	15	31.9	32	40
Female	16	48.5	32	68.1	48	60
**Currently enrolled at school**						
Yes	26	78.8	10	21.3	36	45
No	7	21.2	37	78.7	44	55
**Education level**						
None	3	9.1	3	6.4	6	7.5
Primary (uncompleted)	2	6.1	24	51.1	26	32.5
Secondary (uncompleted)	28	84.8	20	42.6	48	60
**Occupation**						
Domestic	10	30.3	21	44.7	31	38.8
Student	17	51.5	4	8.5	21	26.3
Fisherman	0	0.0	17	36.2	17	21.3
Trader	2	6.1	3	6.4	5	6.3
Farmer	2	6.1	2	4.3	4	5
Others*	2	6.1	0	0.0	2	2.5
**Marital status**						
Single	28	84.8	34	72.3	62	77.5
Married/living with a partner	5	15.2	13	27.7	18	22.5
**Currently pregnant**						
Yes	1	3.0	3	6.4	4	5
No	16	48.5	44	93.6	60	75
Not Applicable^&^	16	48.5		0.0	16	20
**Migration status**						
Migrant	20	60.6	23	48.9	43	53.8
Non-Migrant (residents)	13	39.4	24	51.1	37	46.3
**Have Children?**						
Yes	7	21.2	6	12.8	13	16.3
No	26	78.8	41	87.2	67	83.8

*Football player and mechanic; ^&^All males.

### Data collection

#### Photovoice

**Figure 2.**
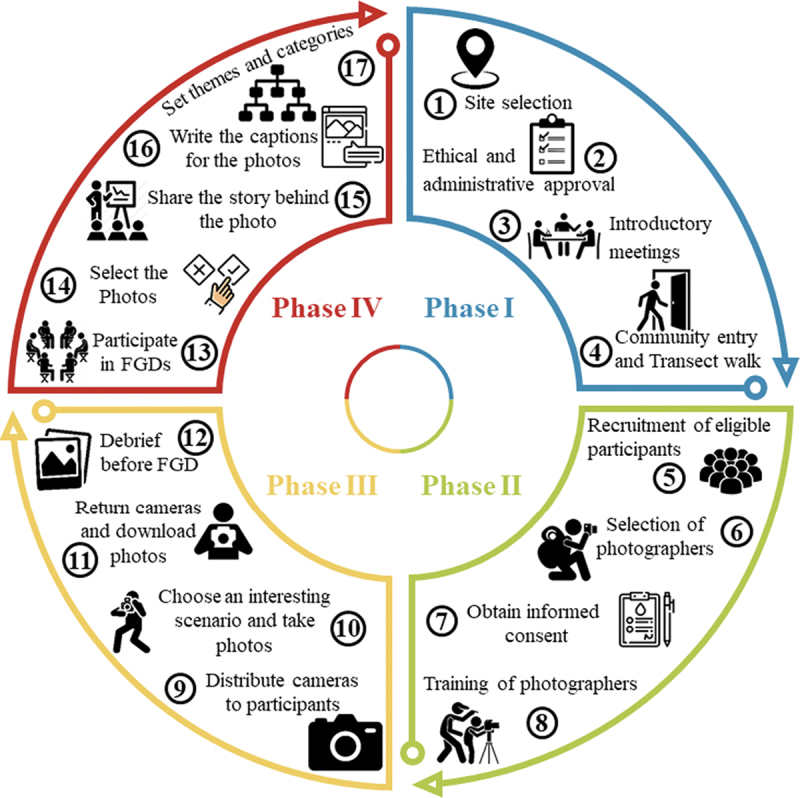
The process adopted by the study to conduct photovoice with adolescents.

In our study, 17 steps were applied to conduct photovoice as illustrated in Figure 2. As a first step, adolescents were visited at home about three days before the FGDs by a male and female research assistant. Caregivers were asked for permission through an informed consent to formally authorise adolescents to actively participate as photographers and/or discussants in the study. After completing the written consent process, those selected as photographers received training through illustrated and verbal instructions. The training covered study objectives and topics of interest for the photos, technical aspects of digital camera use and ethical considerations when taking photos in public spaces and people. Each training session was conducted in a single session, targeting adolescents individually or in groups of three in the presence of their caregivers and the local leader. At the end of each session, participants had the opportunity to ask questions regarding the topics discussed. Field materials (digital camera, charger, and a printout paper with photovoice instructions) were handed over to each adolescent. Within three days of training, photographers were instructed to take 10 photos of their surroundings within three days after the training. The number of photos and time provided was guided by a recent publication showing that it should allow participants to capture, tell a story and provide insights into their lives. After completing the exercise, the photographers met with the local research team an hour before the FGD to upload the photos onto a study computer and return the cameras and accessories. Out of the submitted photos, each photographer was encouraged to identify the three photos they felt were most representative of the impact (positive and negative) of mining on their health and well-being. Overall, the whole process of photovoice, from step 1 to 17 (phase 1 to 4) lasted between 10 and 14 days in each site (Figure 2). Of note, our study adopted the original method recommended by Wang and Burris [2]. Hence, our study did not intend to focus on stimulating policy change, but rather engage adolescents and understand the experiences captured through their lens, specifically related to the most pressing health and well-being issues in mining areas that are meaningful to them.

#### Focus group discussion

A total of eight FGDs were conducted, including two FGDs with adolescent resident girls without children/not pregnant, two with adolescent resident boys, two with adolescent resident girls who are pregnant or mothers of children and two with adolescent migrant boys (Table 2). The time and place for the FGDs were suggested by the research assistants and agreed with the participants in advance. Typically, FGDs were held in a public places (e.g. schools, churches, and community halls).

After convening the group, participants were reminded of the purpose of the FGD and basic rules for the group discussion were introduced. The informed assent form was read out loud to the participants in the language of preference of each participant and explained in a culturally acceptable way to avoid any misinterpretation. Adolescents were invited to ask questions to ascertain comprehension before they signed the assent form; illiterate participants provided a fingerprint to substitute as a signature in the presence of a witness. Participants were also informed about their right to withdraw from the study at any time or to refuse to answer any question that could make them uncomfortable.

The FGDs were facilitated by two research assistants; one moderated the session and one acted as an observer, taking notes of group dynamics and non-verbal communication. The FGDs were oriented by a semi-structured guide, with mostly open-ended questions, organised in a logical sequence (from general to specific), allowing some participant-driven expansion of the ideas to be discussed (supplementary file 1). The guide was structured around five thematic sections: (i) knowledge of diseases in the community, (ii) health seeking behaviour, (iii) barriers to access health care, (iv) photovoice, and (v) perceived impact of mining on health. A separate guide was developed for the photovoice discussion, following the methodology of Wang and Burris [2]. The photos captured by the three photographers served as the basis to generate a discussion on the topics from the participants’ own views on the mining activities on their health and well-being. The photovoice discussion followed the SHOWeD technique to allow participants to describe and reflect on their selected photographs [12]. After selecting the three photos that most represent their experience, photographers were encouraged to explain to the rest of the group the reasons for their choices and how they relate to adolescent health and well-being. Subsequently, the other participants were encouraged to react, comment, discuss, and add their own views on the points illustrated by the photos.

The FGDs were audio-recorded and lasted between 60 and 120 minutes. During the FGDs, detailed field notes were taken to annotate participants’ views and comments to capture group dynamics and non-verbal expressions, and a report was elaborated by the note-taker. All FGDs were conducted in the local or the official language preferred by the participants.

### Data management and analysis

The process of managing, analysing, and interpreting the photos started during the FGDs. Following the Wang and Burris methodology [12], the interactive approach with the participants consisted of three stages: (i) selecting (photos chosen by adolescents that most accurately reflected their needs and views), (ii) contextualising (adolescents explaining the meaning of the photos), and (iii) coding (based on adolescents identifying themes and categories emerging from the meanings of the photos).

All FGDs were digitally recorded and transcribed in *verbatim* and simultaneously translated from the local language to Portuguese by the transcribers. In order to ensure consistency and accuracy, the most experienced team member (senior transcriber-encoder) performed quality control by checking the audio with the transcription to identify and amend any errors. To protect participant privacy, no identifying information (e.g. name) was included in the transcripts and photos that contained identifiable information were anonymised using blurring techniques and pseudonyms during data analysis. All transcriptions were uploaded into NVivo version 12© (QSR International Pty Ltd) to facilitate the organisation and coding of transcripts and photographs.

The FGD transcripts underwent thematic analysis combined with discourse analysis, following an inductive approach, which consisted of initially reading all the transcripts and identifying emerging themes relevant to the study objectives, which subsequently contributed to further shape the analytical plan. The themes were further coded into sub-themes that were linked to categories and subcategories in accordance with the identified patterns. The presentation of the qualitative data complied with the COREQ guidelines for reporting qualitative research [42].

## Lessons learned and discussion

To the best of our knowledge, this is the first paper that critically discusses the experience gained from the application of the photovoice method to understand adolescents’ perceptions about the impact of mining projects on their health and well-being in rural areas in Mozambique. This work will reflect on the lessons learned about the challenges and opportunities of using the photovoice method, in the light of future public health research and interventions in similar contexts. By exploring the potential of capturing adolescent perspectives through photography, we also put the key findings in the context of impact assessment-related research to identify better ways to engage and address the unique health and well-being issues and needs of adolescents.

### Exploring adolescent perspectives through photography

**Figure 3.**
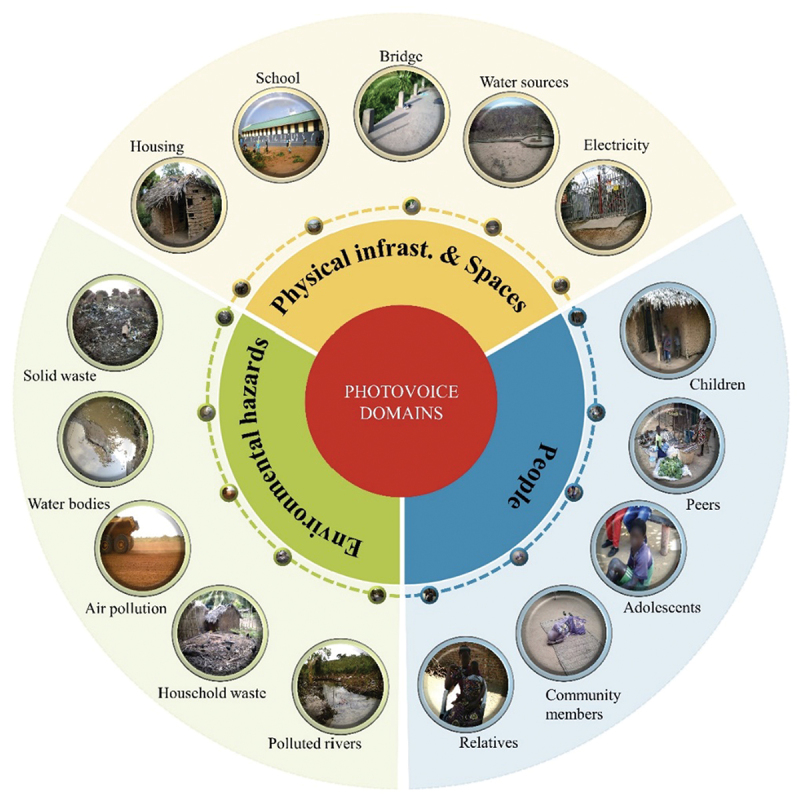
Main domains presented in the photos of adolescents.

The 24 photographers returned 484 photos (384 from Moma district and 100 from Moatize district) and three videos. In summary, the photos considered important by the adolescents displayed three main domains: (i) infrastructure and physical spaces (e.g. hospitals, housing, roads, bridges, schools, churches, energy sources, water sources), (ii) environmental hazards (e.g. solid waste, waste water, dust, polluted air, rivers, and other water bodies), and (iii) people (e.g. relatives, peers, and other community members) (Figure 3).

Altogether, the photos belonging to the ‘infrastructure and physical spaces’ domain were most frequently represented, with 236 (49%) photos, followed by ‘environmental hazards’ with 153 (32%) photos. Within the ‘infrastructure and physical spaces’ domain, the photos tended to focus mostly on ‘housing and road conditions’, while in the domain of ‘environmental hazards’, the most frequent topic was ‘solid waste, waste water, and polluted air’ within the neighbourhood, with 95 photos. Under the ‘people’ domain, photos focused on individuals (children, adolescents, and adults) in their leisure time at home or in the neighbourhoods, with 48 photos.

While some of the themes identified and discussed by the adolescents have been previously documented in other environmental health research in an urban context [43–45], our study added the benefit of a more structured categorisation. This approach helped to identify, categorise, and conceptualise the different aspects that affect the health and well-being of adolescents in mining areas in a more structured way.

The findings of our study showed that infrastructure and physical spaces as well as environmental hazards were the dominant themes represented in the photos. Through photovoice, the environmental and community impacts resulting from the presence of mining projects can be visually presented through photographs. This suggests that photovoice could be a suitable method for studies related to environmental health regardless of the context. In addition, there is potential for further expansion of its use in health impact assessments (HIA) in vulnerable and/or marginalised groups. This is particularly relevant in remote settings, where inequalities prevail, particularly when engagement of adolescents is required. In this context, the inclusion of the photovoice method with vulnerable and/or marginalised groups, including adolescents, can be useful to ensure that the voices and experiences of these stakeholders can be incorporated into the decision-making process on issues that affect them. Additionally, targeted interventions can be tailored to their specific age groups and their unique needs, and sustainable health outcomes are promoted [46].

However, compared to its ability to capture perceptions of physical and environmental aspects affecting adolescents’ well-being, the method was less straightforward in revealing their concerns regarding social, relational, and community aspects that are less tangible. This finding was also reported by researchers that conducted photovoice to document and examine environmental change and injustice within indigenous communities, who attributed this challenge to the nature of visual research methods [14]. Our findings are in line with Ronzi et al. [47] who have also observed in their study that participants have difficulties in taking photographs of negative social concepts (e.g. social isolation) compared to rather than negative physical aspects (e.g. rubbish in the street) which were easily captured [47]. Additionally, the authors reported difficulties in the discussion of ideas that could not be expressed through photographs, as the method was shown to be less appropriate for providing insights beyond the given visual representation of the phenomena [14,15,47].

Our study also found some aspects that surprisingly did not feature very prominently in the photos. For example, although participants expressed concerns about risky sexual behaviours among adolescents (early sexual activity, unsafe sex, transactional sex) in these mining areas, they did not bring any photos that illustrated these scenarios for the photovoice discussion. In addition, ‘adolescent hang out places and leisure activities’ and ‘mining fields’ did not feature among the three photos selected to discuss in the group. This could be attributed to cultural taboos surrounding sensitive topics or lack of visual representation of these issues in their daily lives. It is noteworthy that studies using photos as data often focus solely on what is seen, not raising the question about what remains unrepresented [5]. Therefore, despite our effort to use a visual source of data, a number of questions are left unanswered by our analysis, such as: ‘what type of information, and to what extent, do the photographs produced by adolescents in mining areas offer information that cannot be captured by other methods? What aspects are not visible and require further description/interpretation?’. The use of photovoice as a standalone research method may have limitations [43,44]. While the photos provide an illustration of the lives of adolescents in mining areas, it is only through their verbal sharing of their individual experiences that we can fully grasp the meaning of their perspectives and lived experiences. These findings are in alignment with previous research that observed that photovoice was well suited in providing insights complementing other research methods, such as interviews and FGDs [19,47,48]. Therefore, there is a need to triangulate photovoice with other qualitative techniques, such as interviews and observations, to gain a deeper understanding of aspects that may not be visually represented in the photographs.

### The added value of photovoice method with adolescents

In our study, photovoice gave the photographers a more active role, in comparison to classic data collection approaches, enabling each participant to focus on their own ideas elicited by their own photos, with little or no influence from other discussants, and thereby providing them the opportunity to influence the research process more strongly and equally. These results are aligned with previous studies that have demonstrated that photovoice ensures that participants are empowered to express their perspectives, experiences, and stories through photography [4,49,50]. Similarly, we have seen in the present study that the guided discussions based on the photos allowed adolescents to freely express and introduce the topics on perceived mining impacts on their health and well-being, without the aid of a predetermined FGD guide. The photographers were given the autonomy to select and share photos that best represented their unique experiences, and they took the lead in interpreting the meanings and stories behind them in a way that was meaningful to them without interference from the FGD moderator, who would only inquire about the meaning behind each selected photo. Such perspectives could have been misunderstood or missed entirely if based on verbal accounts only that would in turn have been interpreted by the researchers alone.

Moreover, adding the photovoice component to the FGDs stimulates photographers’ prepardness to voice their views and understanding of their own world, allowing them to feel more comfortable during the discussions, with minimal influence of dominant voices, of peers and researchers alike, away from the normative discourses that are common in standalone FGDs [51,52]. Through this study, the use of photovoice also helped researchers and moderators, who were adults, to get closer to adolescent language, addressing the criticism regarding unequal power relations between researchers and children and adolescent participants in the research process [53]. These results align with the findings of a previous study that utilised photographs to support research with adolescents, demonstrating that this method enhances the researcher–participant relationship, consequently enriching the quality of the collected data [54]. However, this requires the researcher to be in a constant state of self-awareness of their positionality and reflexivity, while actively engaging adolescents at all stages of the research process [53,55].

To date, the advantages and disadvantages of the combination of classic qualitative methods and innovative visual techniques, such as photovoice integrated in FGDs, have not always been carefully examined, resulting in the adoption of such methods without sufficient critical reflection and rigour [56]. In addition, it is not known to what extent the combination of these methods produce in-depth data to develop new knowledge in specific areas or topics [53]. Of note, in the case of our study, image analysis as such was not conducted, on the one hand, because it was beyond the time limits and the scope of the study, and on the other hand, because the team lacked the expertise to do in-depth image analysis.

### Feasibility of applying photovoice with adolescents in mining areas

While the use of photos in environmental research is an innovative approach to conducting participatory research, interestingly, in our study, the photovoice method was a feasible approach for rural mining areas, as it does not require adolescents to have any prior knowledge of technology or literacy skills. To ensure the effective use of the cameras, it is important to provide comprehensive materials and detailed instructions. This approach enabled the photographers to capture the subjective reality and clearly represent their experiences related to their health and well-being in mining areas.

In this study, we found that adolescents were not familiar with the concept of ‘health impacts’. To address this, photographers were oriented to capture both ‘positive’ and ‘negative’ effects of mining through their photos. However, not all of the five SHOWeD questions were included during the discussion when adolescents were asked to talk about the selected photos. Specifically, the questions related to ‘why does this problem or this strength exist?’ and ‘what can we do about this?’ were not proactively used to frame the adolescent story. Instead, the discussion was mostly framed around the following questions: ‘what do you See here?’, ‘what’s really happening here?’ and ‘how does this relate to our lives?’ This was due to current life situation and hence needed to minimise expectations. This is particularly relevant in mining areas, where expectations and desires regarding the presence of mining are common and can be difficult to manage [57]. The study highlighted the need to adapt SHOWeD questions and simplify the photovoice process while minimising expectations in studies conducted in mining settings. Previous research on photovoice conducted with adolescents has also reported modifications of the SHOWeD based on the specific purpose of the projects, such as rewording, reducing, or adding questions [1].

Of note, none of the photographers delivered the exact number of photos requested (10), with the majority delivering more than expected. However, despite the variation in the number of photos, a similar pattern of themes was observed regardless of the number of photos presented by each photographer. This suggests that limiting the number of photos to a maximum of 10 photos and selecting three per participant for discussion is sufficient to reach saturation of themes related to the study topic. These findings align with what has been documented in the wider context of qualitative health research studies [58]. While Wang and Burris recommended the selection of a small set of photographs for discussion and reflection [2,12], we found no recommendations for limiting the number of photos or the number of photographers per session. Two recent reviews conducted by Catalani and Fountain [1,15], evidenced that for photovoice the number of photos and participants varied across different projects based on the specific goals and objectives of the projects, as well as the preferences and capabilities of the participants. Thus, most importantly, the captured images should tell a story, evoke emotions, and provide insights into both the participantsʼ lived experiences and the issue they are addressing [15,48]. In our case, the number of photos was determined by the estimated time that would allow enough discussion with six and three photographers in each session. Therefore, our results suggest that future studies may consider setting a clear limit on the number of photos for participants to simplify data management and analysis without sacrificing the quality of their photovoice study. This could potentially reduce the time needed to collect the data, reduce the cost, and facilitate a more in-depth discussion about the experience of adolescents. This can also assist in finding the right balance between the number of photographs and photographers in each discussion.

The use of digital cameras proved to be a valuable tool in this study. Interestingly, adolescents showed great enthusiasm and interest in participating in the study, which made it easy to attract and engage participants from this age group in research, especially in rural mining areas where participation is expected to be lower compared to urban areas due to cultural and religious issues, security concerns, as well as considering adolescents potential shyness towards using cameras [59]. Unlike standalone FGDs, where adolescents typically act as respondents only, the photovoice method in our study stimulated curiosity and interest in the photos produced. Hence, it stimulated and encouraged adolescents to engage in a reflective process during the FGD sessions, ultimately resulting in more active involvement and interest in the research process.

The openness of the adolescents towards the use of photovoice can be primarily explained by the developmental life stage of adolescence, both cognitive and affective, when adolescents are biologically, emotionally and developmentally primed to develop new skills [60,61] and the increased experience and affinity with digital technology and access to social media by young people in their daily life [62,63]. This observation is consistent with a recent review that concluded that digital technologies could be powerful tools in promoting sexual health but are currently underutilised [63]. In addition, previous studies have also demonstrated that adapting data collection strategies to technological advancements has become essential in conducting health-related research with this age group and encourage adolescentsʼ interest, thought, and enjoyment [62,64,65]. However, our results differ from a previous study, which reported difficulties in involving adolescents in research due to age-related challenges, such as difficulty in verbally expressing themselves, particularly their emotions [55]. Our study highlights the potential of using photovoice and other digital technologies to engage adolescents in public health research, particularly in remote settings where access and use may be limited [1,49,62,66]. There is also a need to invest time in a careful preparation phase, which involves an intensive effort to inform and engage community leaders and caregivers, and the adolescents themselves. Our study reinforces the importance of actively involving influential and trusted community actors throughout the research process and adapting the recruitment strategy in a culturally appropriate way [67,68].

Finally, it is important to acknowledge that our study was conducted in specific rural settings of North and Central Mozambique, inhabited mainly by low-resource vulnerable and/or marginalised populations. The feasibility of integrating the photovoice method with established data collection approaches was critically assessed in accordance with existing theories and guidelines. The results of this study may not be generalisable to other adolescents in mining areas and may only reflect the experiences and perspectives of this particular group of participants. Additionally, the analysis was limited to the photovoice integrated into FGD and a sample of participants that we had access to, and there may have been other important factors that were not captured through this method.

## Conclusion

In this study, the photovoice method has been successfully applied to gain a better understanding of adolescents’ perceptions of the impacts of mining projects on their health and well-being in rural areas in Mozambique. Several lessons for guiding future research in similar settings were learned. First, photovoice allowed adolescents’ active engagement and interest in the study. Second, discussions guided by the photos encouraged adolescents to introduce the topics, freely express themselves, and reflect on their unique experiences and concerns. These were mainly linked with infrastructures/physical spaces and environmental hazards. Finally, compared to its ability to capture perceptions of physical and environmental aspects affecting adolescents’ well-being, the method was less straightforward in revealing their concerns regarding the method was not suitable for capturing social, relational, and community aspects that are less tangible. Nevertheless, there is potential for further expansion of its use in HIA in vulnerable and/or marginalised groups, offering an entry point for future validation in the field of environmental research targeting adolescents. In addition, programmes and interventions can make use of this method to address health issues without setting adolescents’ views and priorities aside, allowing them to influence health and well-being decisions on issues that are meaningful to them while taking into account differences among these groups. Future studies should explore strategies to minimise the role of the existing inequalities, social and cultural positions of adolescents, and pre-existing power dynamics that still affect their engagement and contribution to advocating for necessary and meaningful changes.

## Supplementary Material

declarationStatement.docx

## Paper context

Photovoice is a method used to elicit participantsʼ perspectives through visual representations, empowering vulnerable and marginalised populations. However, no studies have been found that critically reflect on this innovative method to gain insight into adolescentsʼ perspectives in mining areas. A reflection on lessons learned in applying the photovoice method to understand adolescents’ perceptions about the impact of mining projects on their health and well-being in rural areas in Mozambique is discussed.
