# Supplementary material for: Applying the photovoice method with adolescents in mining areas in rural Mozambique: critical reflections and lessons learned
Source: Glob Health Action. 2024 Feb 7;17(1):2305506. doi: 10.1080/16549716.2024.2305506 (PMC10851835; doi:10.1080/16549716.2024.2305506)
Supplement: declarationStatement.docx [file ZGHA_A_2305506_SM0982.docx]

**Declaration of interest**

The authors declare that they have no known competing financial interests or personal relationships that could have appeared to influence the work reported in this paper.
 

 
 
 
